# Investigation of Thermal Deformation Behavior in Boron Nitride-Reinforced Magnesium Alloy Using Constitutive and Machine Learning Models

**DOI:** 10.3390/nano15030195

**Published:** 2025-01-26

**Authors:** Ayoub Elajjani, Yinghao Feng, Wangxi Ni, Sinuo Xu, Chaoyang Sun, Shaochuan Feng

**Affiliations:** 1 School of Mechanical Engineering, University of Science and Technology Beijing, Beijing 100083, China; ayoub@xs.ustb.edu.cn (A.E.); fengyh@xs.ustb.edu.cn (Y.F.); niwangxi@xs.ustb.edu.cn (W.N.); xusinuo@xs.ustb.edu.cn (S.X.); 2 Beijing Key Laboratory of Lightweight Metal Forming, Beijing 100083, China

**Keywords:** thermal deformation behavior, boron nitride-reinforced magnesium composite, support vector regression (SVR), flow stress prediction, machine learning models

## Abstract

Accurate flow stress prediction is vital for optimizing the manufacturing of lightweight materials under high-temperature conditions. In this study, a boron nitride (BN)-reinforced AZ80 magnesium composite was subjected to hot compression tests at temperatures of 300–400 °C and strain rates ranging from 0.01 to 10 s^−1^. A data-driven Support Vector Regression (SVR) model was developed to predict flow stress based on temperature, strain rate, and strain. Trained on experimental data, the SVR model demonstrated high predictive accuracy, as evidenced by a low mean squared error (MSE), a coefficient of determination (*R*^2^) close to unity, and a minimal average absolute relative error (AARE). Sensitivity analysis revealed that strain rate and temperature exerted the greatest influence on flow stress. By integrating machine learning with experimental observations, this framework enables efficient optimization of thermal deformation, supporting data-driven decision-making in forming processes. The results underscore the potential of combining advanced computational models with real-time experimental data to enhance manufacturing efficiency and improve process control in next-generation lightweight alloys.

## 1. Introduction

With the growing demand for lightweight and high-strength materials in various industries, magnesium alloys have gained significant attention. These alloys are valued for their low density, excellent machinability, and good mechanical properties, making them ideal for applications in the automotive and aerospace sectors [1,2,3]. Magnesium, being one of the lightest structural metals, offers significant weight reduction advantages, which translates into improved fuel efficiency and performance. However, the performance of magnesium alloys at elevated temperatures poses challenges, limiting their wider application where thermal stability is crucial. To enhance the high-temperature performance and mechanical properties of magnesium alloys, researchers have explored the addition of reinforcement materials, demonstrating that magnesium alloys reinforced with rare earth elements show good results in biomedical applications [4]. However, the use of two-dimensional (2D) materials such as boron nitride (BN) as reinforcement to enhance the hot deformation behavior of magnesium alloys remains underexplored [5,6]. BN is known for its excellent thermal stability and mechanical strength, presenting a promising option for reinforcement [7]. Such distinctive attributes could contribute to improvements in wear resistance, microstructural stability, and high-temperature performance in alloys like AZ80. As a result, an AZ80 alloy reinforced with BN may hold promise for advanced industrial applications where precise control of material properties is essential.

Understanding and accurately predicting the flow stress during thermal deformation is essential for optimizing manufacturing processes such as forging, rolling, and extrusion [8]. Traditional modeling methods often struggle to capture the complex, nonlinear relationships between process parameters like temperature, strain rate, and material composition [9]. This limitation highlights the need for advanced, data-driven approaches to model the thermal deformation behavior of novel composite materials such as AZ80-BN. Machine learning techniques, particularly Support Vector Regression (SVR), offer powerful tools for modeling complex material behaviors without relying on predefined equations [10]. In a comparative study involving constitutive equations, neural networks, and SVR for modeling the hot deformation of 316L stainless steel, researchers confirmed that SVR can handle multidimensional data and capture nonlinear relationships, making it well-suited for predicting flow stress based on multiple influencing factors.

By integrating machine learning with experimental observations, this research provides a novel approach to understanding and predicting the thermal deformation behavior of AZ80-BN magnesium composite. The developed SVR model not only enhances the accuracy of flow stress predictions but also contributes to the field of intelligent manufacturing by enabling data-driven optimization of forming processes. This work represents the first investigation into the thermal deformation behavior of AZ80-BN composite using SVR, offering valuable insights for future research and industrial applications.

## 2. Experimental Methodology and Materials

**Figure 1:**
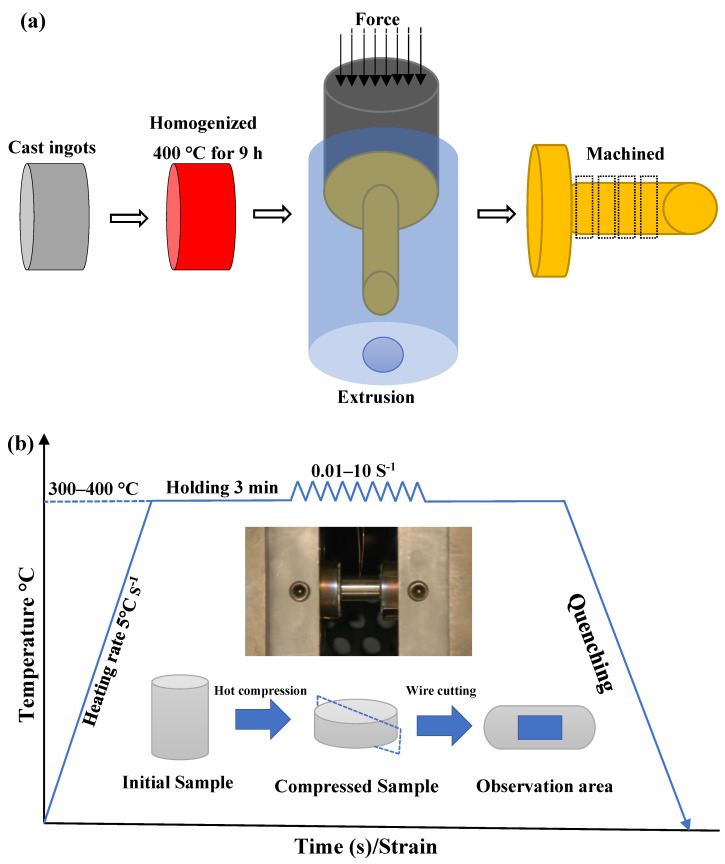
(**a**) Sample preparation process, (**b**) testing workflow for AZ80-BN composite.

The BN-reinforced magnesium composite used in this study was synthesized by incorporating BN particles into the AZ80 magnesium alloy. The BN particles used as reinforcement in the AZ80 magnesium composite were supplied by a commercial source, with a purity of 99.99%. The average particle size was approximately 5 μm, confirming their microscale nature. This microscale size facilitates a uniform dispersion within the AZ80 matrix, enhancing the composite’s mechanical and thermal properties. The high aspect ratio and thermal stability of these BN microparticles contribute significantly to the observed improvements in peak stress and microstructural stability under elevated temperatures. The base alloy was melted in a resistive electric furnace under a protective argon gas mixture to prevent oxidation. Once the alloy reached the melting temperature of 720 °C, BN particles were introduced and thoroughly stirred to ensure homogeneous dispersion within the composite. The molten composite was poured into a steel mold and allowed to cool to room temperature. The resulting ingots were divided into cylindrical specimens measuring 17 mm in diameter and 30 mm in height. These specimens underwent a solution treatment at 400 °C for 9 h, followed by aging at 200 °C for 30 min, and were subsequently air-quenched to room temperature in preparation for hot extrusion. A graphite lubricant was applied to both the billet and the die to minimize friction and prevent wear during the extrusion process. An extrusion ratio of 4.52:1 was used to ensure optimal mechanical properties and a uniform microstructure. Finally, cylindrical samples measuring 8 mm in diameter and 10 mm in height were machined from the extruded material for compression testing. Figure 1a provides a schematic of the mold structure and the process workflow, from casting to machining. The chemical compositions (wt.%) of the experimental materials are as follows: AZ80 alloy: Al 8.16%, Zn 0.42%, Mn 0.30%, balance Mg; and AZ80-BN composite: Al 4.55%, Zn 0.25%, BN 0.51%, Mn 0.18%, balance Mg.

Hot uniaxial compression tests were conducted using a Gleeble thermomechanical simulator. The deformation temperatures ranged from 300 °C to 400 °C, and the strain rates varied between 0.01 s^−1^ and 10 s^−1^. To minimize friction during testing, graphite lubricant sheets were applied to both ends of the samples. The specimens were inductively heated to the desired deformation temperature within 30 s and held at this temperature for 3 min to ensure uniform thermal distribution prior to deformation. Compression was carried out until a true strain of 0.65 was achieved, followed by immediate water quenching to room temperature to preserve the deformed microstructure. Force–displacement data were recorded during testing, and true stress–true strain curves were generated from these measurements. Dimensional measurements of the deformed samples were also taken to validate deformation consistency. Each test condition (combination of temperature and strain rate) was repeated three times to ensure the reliability and reproducibility of the results. The average values from these repetitions were used for analysis. Additionally, the hot uniaxial compression tests were conducted in accordance with the ASTM E209-18 standard [11], which provides guidelines for compressive stress–strain testing at elevated temperatures.

**Figure 2:**
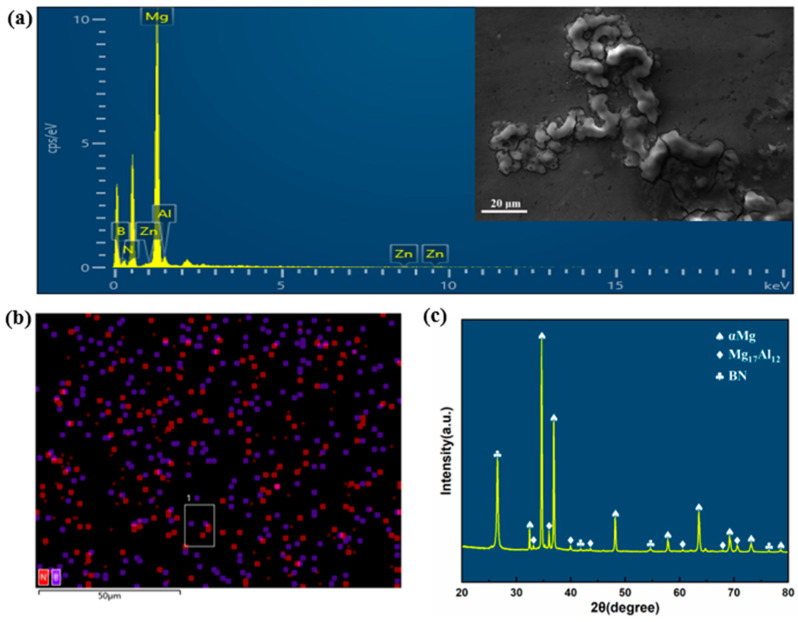
EDS analysis. (**a**) Element spectrum corresponding to AZ80-BN composite. The inset image shows the SEM-secondary electron (SE) scan area used for chemical composition analysis, (**b**) EDS element mapping image, and (**c**) X-ray diffraction patterns.

The compressed samples were cut in half along the compression axis, and the cross-section was taken as the testing surface for microstructure characterization, as illustrated in Figure 1b. Subsequently, the testing surface was polished using 400–2000# sandpapers as well as diamond paste and then etched using a 4 vol% nitric acid alcohol solution. To confirm the incorporation and distribution of BN particles in the AZ80 matrix under specific deformation conditions, a comprehensive microstructural characterization was performed on a sample deformed at 400 °C and 10 s^−1^. Scanning electron microscopy (SEM), energy-dispersive spectroscopy (EDS), and X-ray diffraction (XRD) analyses were performed. The SEM-secondary electron image revealed detailed microstructural features, confirming the successful incorporation of BN particles. The EDS element mapping indicated a uniform distribution of BN throughout the matrix, while the XRD patterns validated the presence of BN phases, confirming their effective integration into the AZ80 matrix. Figure 2 showcases these findings, including the spectral data, element mapping, and diffraction patterns that collectively verify the structural and compositional integrity of the AZ80-BN composite.

## 3. Results and Discussion

### 3.1. True Stress–Strain Curve

**Figure 3:**
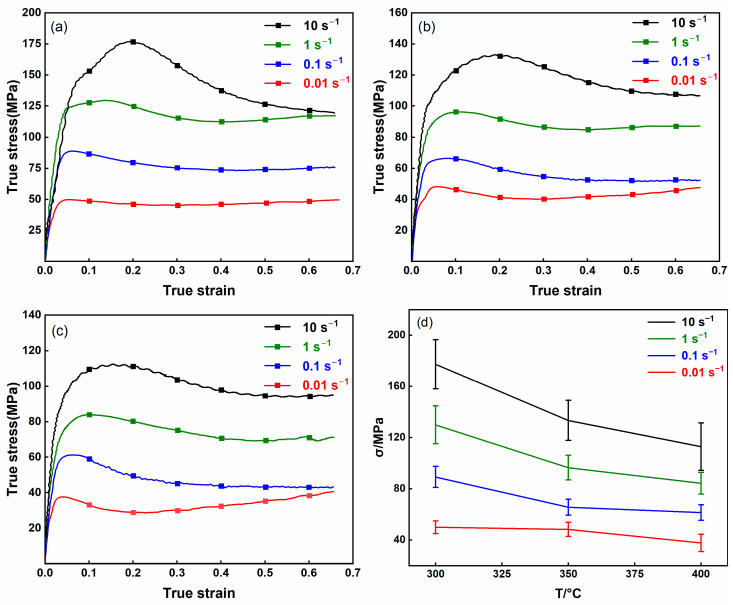
True stress–true strain curves of AZ80-BN magnesium composite under various deformation conditions. (**a**) 300 °C, (**b**) 350 °C, (**c**) 400 °C, and (**d**) peak stress variation with temperature across different strain rates.

The true stress–strain curves obtained from uniaxial compression tests at various temperatures and strain rates are shown in Figure 3a–c. The flow behavior of the AZ80-BN composite is strongly influenced by the deformation temperature and strain rate: as the strain rate increases and the temperature decreases, the flow curves shift to higher stress levels. Based on the evolving stress response and microstructural changes, the deformation behavior can be divided into three distinct regions. Initially, the stress increases sharply to a peak (Figure 3d), primarily driven by pronounced work hardening—consistent with previous reports [12]. This early hardening stage is mainly attributed to dislocation multiplication in the matrix, which elevates dislocation density and enhances the resistance to plastic flow. As deformation proceeds beyond this peak, the stress gradually decreases, signaling the onset of dynamic recovery (DRV) and dynamic recrystallization (DRX). Notably, the incorporation of BN introduces two-dimensional phases that impede dislocation motion and grain boundary migration. Consequently, the peak stress of the AZ80-BN composite exceeds that of the unreinforced AZ80 alloy [13]. Eventually, DRV and DRX processes facilitate dislocation rearrangement and annihilation, thereby offsetting the initial work-hardening effect and promoting flow softening.

### 3.2. Prediction of Rheological Stresses by the Intrinsic Equations

The thermal deformation process of AZ80-BN magnesium composite is a thermally activated process influenced by the deformation temperature and strain rate. In thermoplastic deformation, according to the model proposed by Sellars and McTegart [14], the relationship between flow stress, strain rate, and deformation temperature across different stress levels is described by the following equation:

(1) $$\dot{\varepsilon }=A{\left[\sinh(\alpha \sigma )\right]}^{n}\exp\left(\frac{-Q}{RT}\right)\;(\mathrm{For}\;\mathrm{all}\;\alpha \sigma )$$

(2) $$\dot{\varepsilon }=A_{1}\sigma^{n_{1}}\exp\left(\frac{-Q}{RT}\right)\;(\alpha \sigma \;<\;0.8)$$

(3) $$\dot{\varepsilon }=A_{2}\exp(\beta \sigma )\exp\left(\frac{-Q}{RT}\right)\;(\alpha \sigma \;>\;1.2)$$

where $\dot{\varepsilon }$ represents the strain rate. Under high strain rate conditions, achieving a steady state is challenging, so *σ* is taken as the peak stress (MPa). *Q* denotes the deformation activation energy (J/mol), while *A*, *A*_1_, *A*_2_, *β*, *n*, *n*_1_, and α (α = *n*_1_/*β*, MPa^−1^) are material parameters. *R* is the universal gas constant, and *T* refers to the deformation temperature in Kelvin (K).

By applying the natural logarithm to both sides of Equations (1)–(3), respectively, to obtain Equations (4)–(6):

(4) $$\ln\dot{\varepsilon }=\ln A+n\ln\left[\sinh\left(\alpha \sigma \right)\right]-Q/RT$$

(5) $$\ln\dot{\varepsilon }=\ln A_{1}+n_{1}\ln\sigma -Q/RT$$

(6) $$\ln\dot{\varepsilon }=\ln A_{2}+\beta \sigma -Q/RT$$

**Figure 4:**
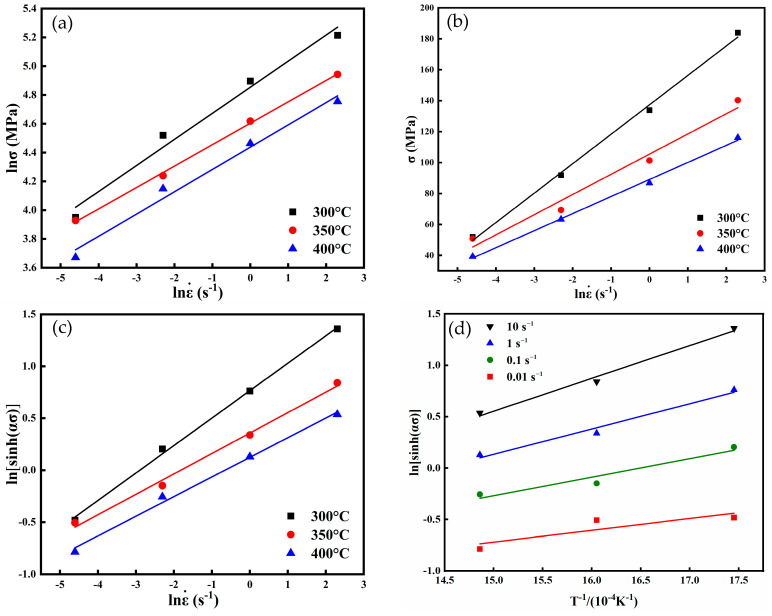
Relations for (**a**) ln*σ* vs. ln$\dot{\varepsilon }$, (**b**) *σ* vs. ln$\dot{\varepsilon }$, (**c**) ln[sinh(*ασ*)] vs. ln$\dot{\varepsilon }$, and (**d**) ln[sinh(*ασ*)] vs. *T*^−1^.

All the aforementioned equations can be generalized into the form of a linear equation, *y* = *ax* + *b*. Based on Equations (5) and (6), the $ln\dot{\varepsilon }-ln\sigma$ and $ln\dot{\varepsilon }-\sigma$ plots were constructed, as shown in Figure 4a,b. The average slopes of these plots were calculated as *n*_1_ = 0.069 and *β* = 6.191, respectively. Substituting the corresponding values of *n*_1_ and *β*, *α* was determined to be 0.011 MPa^−1^. Based on Equation (4) and by fitting $ln\dot{\varepsilon }-ln\left[sinh\left(\alpha \sigma \right)\right]$ in Figure 4c, the average *n* was determined to be 4.620.

The activation energy can be expressed as follows [15]:

(7) $$Q=Rn\left[\frac{\partial \ln[\sinh(\alpha \sigma )]}{\partial \left(\frac{1}{T}\right)}\right]=R\cdot n\cdot K$$

where *K* is the average value of slopes that can be obtained from ln[sinh(*ασ*)] versus 1/*T* plots in Figure 4d. The average value of slope *K* was found to be 0.215, which gave an average value of *Q* as 82.54 kJ/mol. This is lower than the self-diffusion activation energy of magnesium (135 kJ/mol) [16] and other Mg-based alloys, such as Mg-5Li-3Al-2Zn (159.8 kJ/mol) [17] and Mg-9Li-3Al-2Y (95.45 kJ/mol) [18]. The lower activation energy in BN-reinforced AZ80 is due to the BN nanoparticles, which impede dislocation movement and enhance the material’s strength.

Zener and Hollomon jointly proposed a temperature-compensated strain-rate factor *Z* parameter that relates the strain rate to the deformation temperature, which is expressed as [19]:

(8) $$Z=\dot{\varepsilon }\exp\left(Q/RT\right)=A{\left[\sinh(\alpha \sigma )\right]}^{n}$$

Taking logarithms on both sides of Equation (8) can be obtained:

(9) $$\ln Z=\ln A+n\ln\left[\sinh(\alpha \sigma )\right]$$

**Figure 5:**
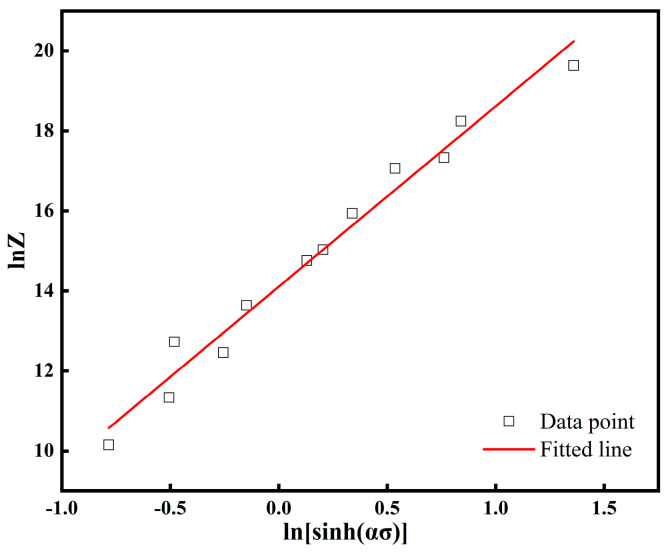
Relation between hyperbolic sinusoidal stress and Zener–Hollomon parameter (*Z*).

The lnZ−ln[sinh(ασ)] plot gives a slope intercept ln*A* = 14.10 and ${A=e}^{14.10}$ (Figure 5).

Finally, the constitutive equation of the AZ80-BN composite can be obtained as follows:

(10) $$\dot{\varepsilon }=e^{14.10}{\left[\sinh(0.0112\sigma )\right]}^{4.620}\exp\left(\frac{-82.54}{RT}\right)$$

The predictive equation for the calculation of true stress is established by rearranging Equation (10):

(11) $$\sigma =\frac{1}{\alpha }\times \sinh^{-1}\left[{\left(\frac{Z}{A}\right)}^{1/n}\right]=\frac{1}{0.0112}\times \sinh^{-1}\left[{\left(\frac{Z}{e^{14.10}}\right)}^{1/4.620}\right]$$

The average absolute relative error (*AARE*) and multiple coefficients of determination (*R*) computed by Equation (12) are used to value the accuracy between the experimental data and fitting results.

(12) $$\left\{\begin{array}{l} R=\sqrt{1-\frac{\sum_{1}^{i=1}{\left(Q_{i}-q_{i}\right)}^{2}}{\sum_{1}^{i=1}{\left(Q_{i}-\bar{Q}\right)}^{2}}}\\ AARE=\frac{1}{n}\sum_{i=1}^{n}\frac{\left|Q_{i}-q_{i}\right|}{Q_{i}}\times 100\% \end{array}\right.$$

where *Q_i_* is the experimental data, *q_i_* is the value calculated by equation, $\bar{Q}$ is the average value of the experimental data, and *n* is the number of data.

**Figure 6:**
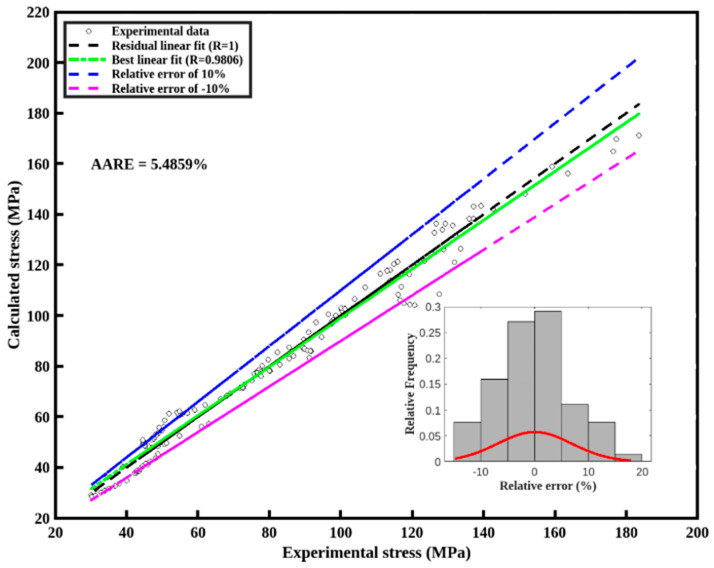
Correlation between experimental and calculated flow stress data.

Figure 6 shows the correlation between the experiment and calculated flow stress data. The results reflect that almost all points are located in the region where the relative error ranges from −10% to 10% and the AARE is 5.48%. These results also confirm that the Arrhenius-type relation demonstrates excellent accuracy for predicting peak stress; however, it may not be suitable for applications requiring strain-related predictions.

**Figure 7:**
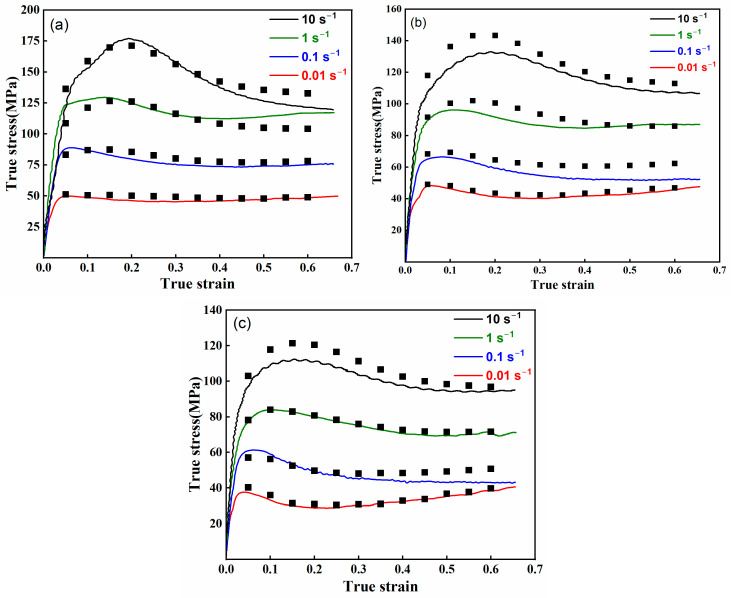
Experimentally measured flow stress (solid lines) vs. Arrhenius model predictions (black squares) at different temperatures: (**a**) 300 °C, (**b**) 350 °C, and (**c**) 400 °C.

The thermal deformation process of the AZ80-BN magnesium composite was modeled using an Arrhenius-type equation to predict flow stress under various deformation conditions. Figure 7 compares the experimentally measured flow stress curves with the corresponding Arrhenius model predictions at different temperatures. The results show that the model achieves reasonable agreement with experimental data, particularly at lower strain rates (0.01 s^−1^ and 0.1 s^−1^), where dynamic recovery (DRV) and dynamic recrystallization (DRX) dominate.

At higher strain rates (1 s^−1^ and 10 s^−1^), slight deviations between the experimental and predicted values are observed, likely due to the model’s inability to fully capture strain-rate-sensitive mechanisms such as dislocation multiplication and grain boundary sliding. Nevertheless, the Arrhenius model accurately predicts the peak flow stress and captures the overall flow-softening trends at elevated temperatures, demonstrating its utility for general flow stress prediction.

### 3.3. Thermal Processing Map

The thermal processing map is an essential tool for optimizing thermal processing techniques, documenting the material’s plastic deformation capabilities under various thermal deformation conditions, including the influence of deformation temperature, strain rate, and strain, obtained by overlaying power dissipation maps and instability maps [20]. The power dissipated by the alloy during thermal deformation consists of two parts: one part is dissipated due to plastic deformation (*G*), and the other part is dissipated due to microstructural changes (*J*) [21], expressed as:

(13) $$P=\sigma \dot{\varepsilon }=G+J=\int_{0}^{\sigma }\sigma d\dot{\varepsilon }+\int_{0}^{\sigma }\dot{\varepsilon }d\sigma$$

The power dissipation factor (*η*) is utilized during the forming stage of materials to depict the ratio of energy consumed during the processing process, which has a close correlation with the microstructural evolution of the alloy [22,23]. Its expression is:

(14) $$\eta =\frac{J}{J_{\max}}=\frac{2m}{m+1}$$

In this equation, *J*_max_ represents the maximum power dissipation, while *m* denotes the strain rate sensitivity index, with the expression for *m* being:

(15) $$m=\frac{\partial J}{\partial G}=\frac{\dot{\varepsilon }\partial \sigma }{\sigma \partial \dot{\varepsilon }}=\frac{\partial \ln\sigma }{\partial \ln\dot{\varepsilon }}$$

The value of *η* does not directly reflect the formability of the material; instead, it needs to be studied in conjunction with instability maps. The formula for the instability map, obtained using the theory proposed by Prasad et al. [24], is as follows:

(16) $$\xi (\dot{\varepsilon })=\frac{\partial \ln(\frac{m}{m+1})}{\partial \ln\dot{\varepsilon }}+m<0$$

where *ξ* ($\dot{\varepsilon }$) represents the instability parameter, and when *ξ* ($\dot{\varepsilon }$) is less than 0, the alloy undergoes flow instability. Utilizing the instability parameter can guide the design and optimization of the processing process.

**Figure 8:**
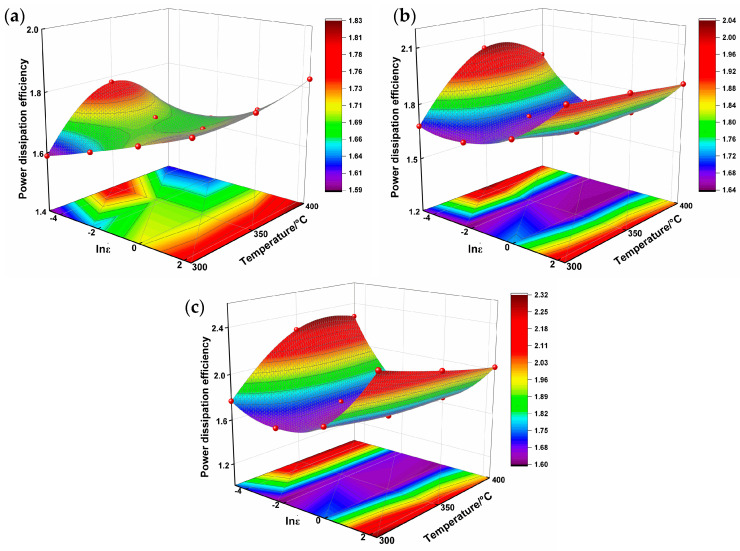
Three-dimensional power dissipation maps of AZ80-BN alloy at different true strains: (**a**) 0.2; (**b**) 0.4; (**c**) 0.6.

A thermal 3D processing map of AZ80-BN was generated by superimposing power dissipation and instability maps at three true strains (0.2, 0.4, and 0.6), as shown in Figure 8. The contour values in these 3D maps represent the power dissipation efficiency (*η*), providing insights into the material’s deformation mechanisms. Regions with higher *η* values indicate enhanced energy dissipation, primarily through mechanisms such as dynamic recrystallization (DRX), whereas highlighted instability zones reveal potential areas of flow localization or defect formation. At a true strain of 0.2, the instability regions are relatively small, suggesting favorable deformation behavior under these conditions. However, when the strain increases to 0.4, these regions expand significantly, indicating a heightened likelihood of defects or localized flow. By contrast, at a strain of 0.6, the instability regions decrease somewhat compared to 0.4, reflecting moderate improvements in material stability. These processing maps also underscore the critical roles of temperature and strain rate in governing AZ80-BN’s deformation response. As the strain increases, the evolving instability regions highlight the complex interplay among strain, temperature, and strain rate in determining alloy workability. Consequently, optimizing these deformation parameters is essential for achieving stable plastic flow and minimizing the risk of instability-related defects.

### 3.4. Machine Learning Approaches

Despite the advancements offered by classical constitutive models in quantifying hot deformation behavior, these models often operate under simplified assumptions and may not fully capture the intricate nonlinearities inherent in complex material systems [25]. As the deformation conditions deviate from standard regimes or the underlying physics becomes too complex to be fully characterized by closed-form equations, machine learning offers a powerful alternative [26]. In this study, Support Vector Regression (SVR) was utilized to enhance rheological stress prediction. SVR was selected for its ability to handle nonlinear relationships effectively, even with limited training data, and for its robustness against noise, ensuring stable and generalized models [27]. The SVR model ingests the same input features (e.g., temperature, strain, strain rate) as classical constitutive models but operates in a data-driven manner. Unlike traditional approaches that rely on curve-fitting procedures to manually determine model parameters, the SVR algorithm learns directly from the training data. This enables the model to adapt flexibly and produce accurate predictions across a wider range of deformation conditions. Subsequent sections will detail the parameter tuning, kernel selection, and training processes for the SVR model. By leveraging this machine learning approach, the study establishes a framework that significantly improves the predictive capabilities for rheological stress in complex deformation scenarios, surpassing the limitations of classical constitutive models.

#### 3.4.1. The Principles of SVR

Support Vector Regression (SVR), introduced by Stitson et al. [28], extends the principles of Support Vector Machines (SVMs) to regression tasks. Built on the foundation of structural risk minimization and statistical learning theory, SVR excels in predicting continuous variables by prioritizing generalization over exact fitting to the training data. Unlike traditional regression methods, SVR introduces an *ε*-insensitive loss function that establishes a margin of tolerance (*ε*) within which predictions are not penalized. This approach enhances the model’s robustness by focusing on significant deviations while ignoring minor fluctuations.

For nonlinear problems, SVR maps input data into a higher-dimensional feature space where linear regression can be applied. This transformation is performed using a mapping function *Φ*(*x*), and the process is represented as:

(17) $$x\overset{\Phi (x)}{\to }\left(\phi_{1}(x),\phi_{2}(x),\ldots \ldots ,\phi_{e}(x)\right)$$

In Equation (1), *x* is the input variable, *Φ*(*x*) is the mapping function, and *e* is the dimension of the transformed feature space. However, computing *Φ*(*x*) explicitly can be computationally intensive. Kernel functions, such as the radial basis function (RBF), implicitly perform this transformation without explicitly computing the mapped features. The RBF kernel is expressed as:

(18) $$k(x_{i},x_{j})=\exp(-\gamma {\left\|x_{i}-x_{j}\right\|}^{2})$$

In Equation (2), *γ* determines the influence of individual data points on the model, balancing flexibility and generalization.

The regression function *f*(*x*) in SVR is defined as:

(19) f(x)=ω·x+b

In Equation (3), *ω* is the weight vector in the feature space, *Φ*(*x*) is the mapping function, and *b* is the bias term. The optimization objective for SVR minimizes the regularization term and the *ε*-insensitive loss function:

(20) $$\underset{\omega ,b}{\min}\left\{\frac{1}{2}{\left\|\omega \right\|}^{2}+C\sum_{i-1}^{n}L_{\varepsilon }(f(x_{i})-y_{i})\right\}$$

In Equation (4), *C* is the regularization parameter, and the loss function $L_{\varepsilon }$ is defined as:

(21) $$L_{\varepsilon }(u)=\left\{\begin{array}{l} 0\to \;\;\;\;\;\;\;\;if\;\;\;\;\;\;\;\left|u\right|\leq \varepsilon \\ \left|u\right|-\varepsilon \;\;\;\;\;\;\to \;\;\;\;\;\;otherwise \end{array}\right.$$

By introducing slack variables $\xi_{i}$ and $\xi_{i}^{*}$, the optimization problem can be rewritten as:

(22) $$\underset{\omega ,b,\xi ,\xi^{*}}{\min}\left\{\frac{1}{2}{\left\|\omega \right\|}^{2}+C\sum_{i=1}^{n}\left(\xi_{i}+\xi_{i}^{*}\right)\right\}$$

subject to:

(23) $$\left\{\begin{array}{l} y_{i}-f(x)\leq \varepsilon +\xi_{i}\\ f(x_{i})-y_{i}\leq \varepsilon +\xi_{i}^{*}\\ \xi_{i},\xi_{i}^{*}\geq 0 \end{array}\right.$$

To simplify the computation, the problem is transformed into its dual form using Lagrange multipliers $\alpha_{i}$ ≥ 0 and $\alpha_{i}^{*}$. The dual form is expressed as:

(24) $$\underset{\alpha ,\alpha^{*}}{\max}\left\{\sum_{i=1}^{n}(\alpha_{i}^{*}-\alpha_{i})y_{i}-\frac{1}{2}\sum_{i=1}^{n}\sum_{j=1}^{n}\left(\alpha_{i}^{*}-\alpha_{i})(\alpha_{j}^{*}-\alpha_{j})k(x_{i},x_{j}\right)\right\}$$

subject to:

(25) $$\sum_{i=1}^{n}\left(\alpha_{i}-\alpha_{i}^{*}\right)=0\Rightarrow \;0\;\leq \;\alpha_{i},\alpha_{i}^{*}\leq C$$

In Equation (8), *K* (*x_i_, x_j_*) is the kernel function, and the solution depends only on the support vectors, which are data points lying on or outside the *ε*-margin.

**Figure 9:**
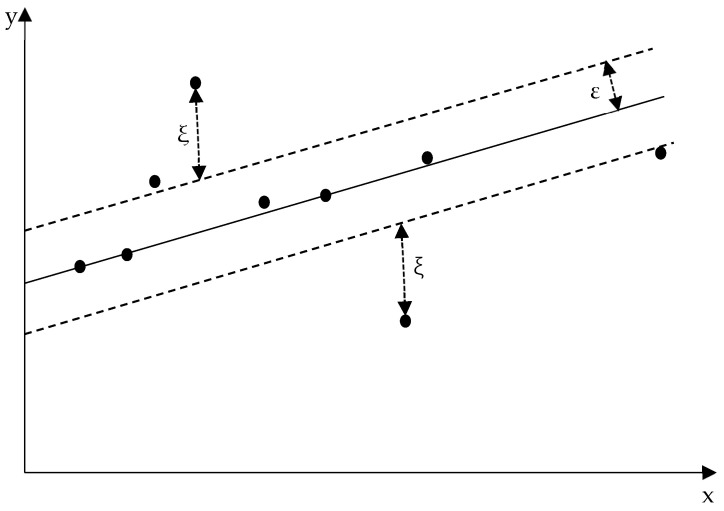
Support Vector Regression, showing the *ε*-margin, slack variables, and hyperplane fitted by SVR.

Figure 9 illustrates the SVR concept, highlighting the *ε*-margin, support vectors, and slack variables. Data points within the margin are ignored by the loss function, while those outside contribute to the optimization objective.

SVR’s ability to combine flexibility, robustness, and scalability makes it ideal for modeling nonlinear relationships in complex systems [29]. In this study, SVR is applied to predict the flow stress of AZ80-BN magnesium composite during thermal deformation, with strain, strain rate, and temperature as input variables. By leveraging the *ε*-insensitive loss function and kernel transformations, SVR enables precise and scalable predictions, which are crucial for optimizing material processing.

#### 3.4.2. Model Performance

In the previous section, we utilized the hyperbolic sine function model to mathematically describe and predict the true stress–strain curve obtained from hot compression tests. However, it is important to acknowledge that twinning deformation during hot compression significantly impacts the alloy’s behavior [30]. To address this limitation and more accurately capture the material’s response, we developed a model that precisely describes and predicts the hot deformation characteristics of the alloy.

Support Vector Regression (SVR) was selected for this purpose due to its powerful capabilities in modeling and predicting continuous target variables, along with its adaptability and strong generalization performance. By inputting the alloy’s temperature, strain rate, and strain into the SVR model and applying an appropriate kernel function, the model generates the flow stress of the alloy as the output. The training data for the SVR model consisted of temperatures ranging from 300 °C to 400 °C in 50 °C increments, strain rates from 0.01 s^−1^ to 10 s^−1^, and strains from 0 to 0.85. The dataset was partitioned with 80% used for training the model. To ensure a robust evaluation and prevent high variance in the results that can occur with a single train-test split, we employed 5-fold cross-validation. This approach provides more reliable *R*^2^ values and offers a more robust estimate of performance by testing the model on different subsets, thereby enhancing its ability to generalize across multiple data subsets. To select the optimal random state for the kernel models, we tested 11 different random states and compared them using the mean squared error (MSE), as shown in Table 1. Controlling the random state in the selection of the training set ensures that results are consistent and reproducible. The performance of the SVR model in terms of prediction and fitting was evaluated using the coefficient of determination (*R*^2^) and mean squared error (MSE) [31]. By meticulously tuning the model parameters and employing rigorous validation techniques, we ensured that the SVR model accurately captures the complex relationships influencing the flow stress of the AZ80-BN magnesium composite during thermal deformation. This approach addresses the limitations of traditional models and provides a more reliable tool for predicting material behavior under various processing conditions.

**Figure 10:**
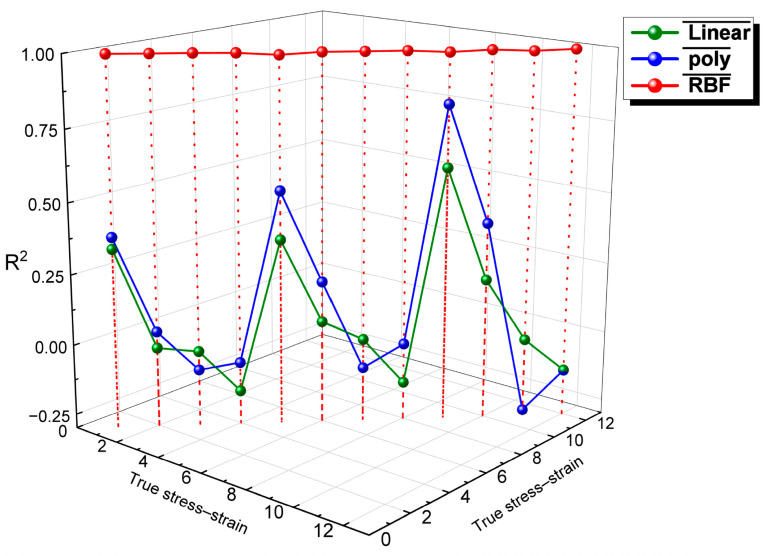
Comparison of *R*^2^ values for linear, polynomial, and RBF kernels.

Figure 10 presents a comparison of the *R*^2^ values for three kernel functions (linear, polynomial (poly), and radial basis function (RBF)) used in the SVR models applied to the hot compression curves. It is evident from the figure that the RBF kernel function yields the highest *R*^2^ coefficient among the 12 true stress–strain curves. This indicates that the RBF kernel function is the most suitable for describing and predicting the hot deformation behavior of the AZ80-BN magnesium composite.

**Figure 11:**
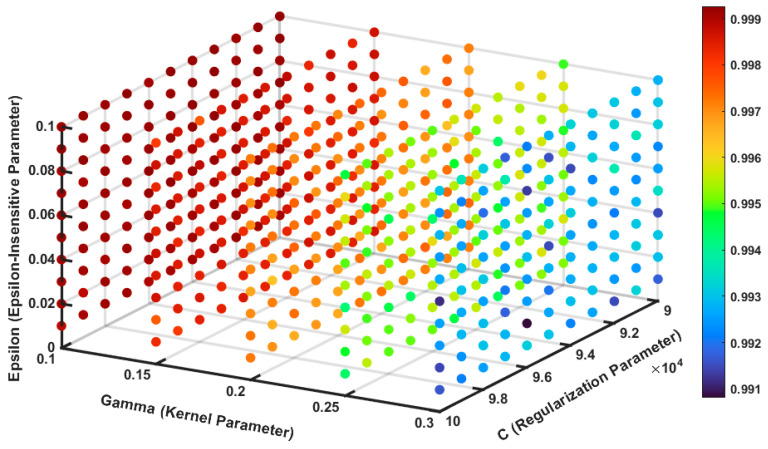
Three-dimensional heat map for *R*^2^ correlation analysis.

During the parameter adjustment process, only the values of the penalty factor (*C*), the *ε*-insensitive loss function, and the kernel parameter (gamma, *γ*) need to be adjusted to improve the accuracy of the SVR model in describing and predicting the thermal deformation behavior [32,33,34]. By utilizing the 3D grid search method, we identified the optimal combination of *C*, *ε*, and *γ* through the *R*^2^ correlation analysis heat map shown in Figure 11, which maximizes the *R*^2^ coefficient of the SVR model for curve fitting [35].

Initially, we set the range of *C* values from 1000 to 100,000 with a step size of 1000, the range of *γ* values from 1 to 11 with a step size of 1, and the range of *ε* values from 0.1 to 1 with a step size of 0.1. However, we observed that increasing *C* and testing lower values of *γ* and *ε* could potentially improve the *R*^2^ value further. Therefore, we optimized the range for *C* from 90,000 to 100,000 in steps of 1000 and refined the *γ* range to 0.1 to 0.3 in steps of 0.05 and *ε* range from 0.01 to 0.1 in steps of 0.1, focusing on smaller *γ* and *ε* values. The final optimal combination obtained was *C* = 95,000, *ε* = 0.1, and *γ* = 0.1 with best *R*^2^ = 0.9993. This combination enabled the SVR model to achieve the highest *R*^2^ coefficient in curve fitting, indicating superior predictive performance.

By fine-tuning these parameters, the SVR model effectively captures the complex nonlinear relationships inherent in the hot deformation process of the AZ80-BN magnesium composite. The use of the RBF kernel with the optimized parameters significantly enhances the model’s ability to predict flow stress under various thermal deformation conditions, contributing to more accurate and reliable process optimization in manufacturing applications.

#### 3.4.3. Validation of the Model

**Figure 12:**
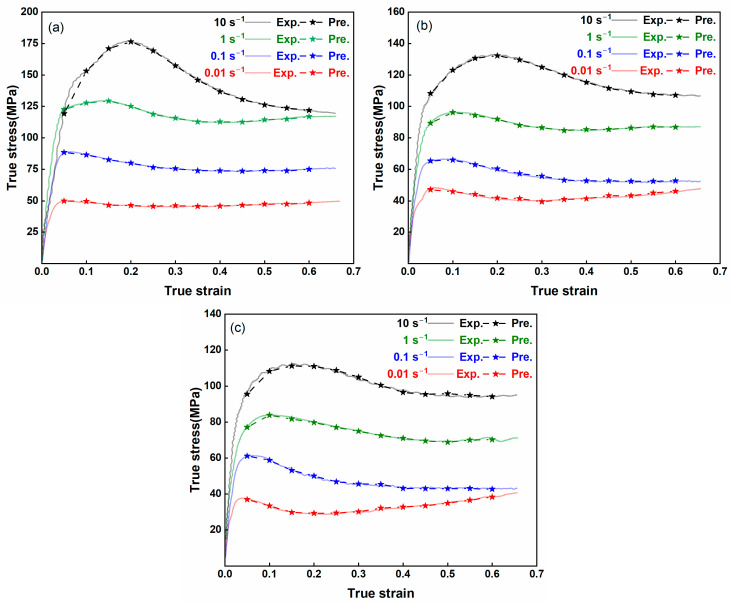
Comparison of experimental (Exp) and SVR model (Pre) flow stress predictions across various strains, strain rates, and temperatures at (**a**) 300 °C, (**b**) 350 °C, and (**c**) 400 °C.

**Figure 13:**
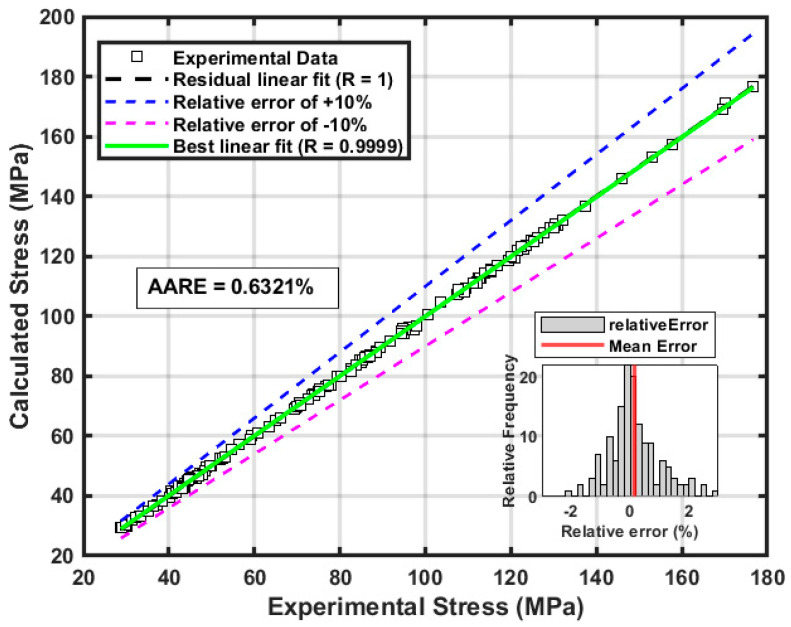
Comparison of the correlation and average absolute relative error between predicted and experimental flow stress values for the AZ80-BN magnesium composite.

Figure 12 compares the experimentally measured flow stress (“Exp”) with the flow stress predicted by the SVR model (“Pre”) across various strains, strain rates, and temperatures. The predicted data closely follow the experimental hot deformation curves, underscoring the SVR model’s high predictive accuracy. As illustrated in Figure 13, a statistical comparison between the predicted and experimental values shows an outstanding coefficient of determination (*R*^2^) of 0.99999 and an average absolute relative error (*AARE*) of 0.6321%. These results highlight the SVR model’s remarkable precision in capturing the thermal deformation behavior of the AZ80-BN magnesium composite.

**Figure 14:**
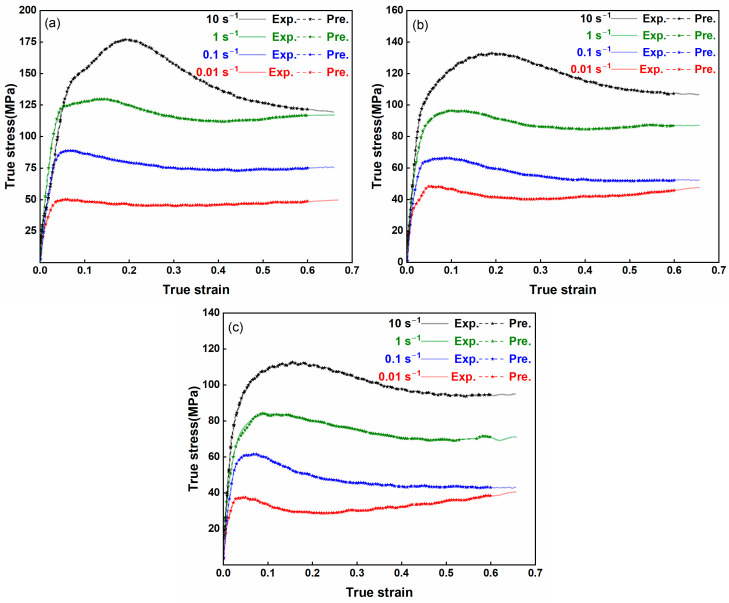
SVR-based flow stress predictions at (**a**) 300 °C, (**b**) 350 °C, and (**c**) 400 °C, evaluated using 110 randomly selected stress points across the strain range.

**Figure 15:**
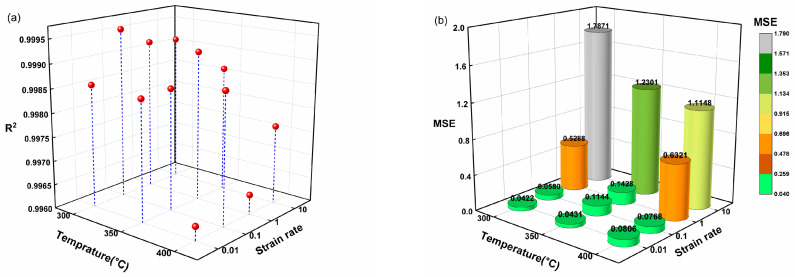
(**a**) *R*^2^ and (**b**) MSE of SVR predictions based on 110 randomly selected stress points spanning the experimental domain.

Previous investigations have often employed uniform step sizes (e.g., fixed increments in strain) for validating predictive models, potentially limiting the demonstration of accuracy across the complete experimental domain [36,37,38]. In this study, we adopted a more comprehensive approach by randomly selecting 110 stress points spanning the entire strain range (0.05–0.6) for model evaluation. Furthermore, we used mean squared error (MSE) instead of *AARE* to evaluate the model’s performance, as MSE emphasizes larger errors and provides a more sensitive measure of absolute prediction accuracy, which is critical for capturing deviations in flow stress predictions. Figure 14 presents the SVR model’s predicted flow stress curve, while Figure 15 provides the corresponding *R*^2^ and MSE values when comparing the predictions with the experimental data under all considered conditions.

As shown in Figure 14, the majority of the predicted stress points closely match the experimental values, indicating excellent agreement. In Figure 15, the average *R*^2^ of 0.9984 and MSE of 0.4876 confirm that the SVR model maintains exceptional prediction accuracy and minimal error levels across a wide range of processing parameters. These findings further validate the SVR model’s effectiveness in representing the thermal deformation characteristics of the AZ80-BN magnesium composite.

Overall, the SVR model demonstrates outstanding predictive capabilities, adeptly handling the nonlinear relationships between rheological stress and processing parameters. Its proven performance and adaptability suggest significant potential for optimizing industrial forming processes involving lightweight materials.

### 3.5. Comparison of the SVR Model with Traditional Methods

**Figure 16:**
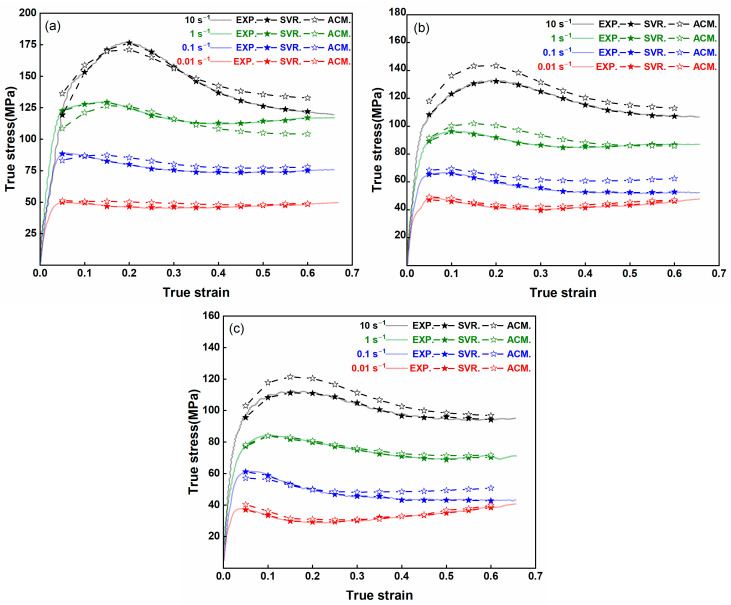
Comparison of EXP, SVR model, and ACM flow stress predictions at (**a**) 300 °C, (**b**) 350 °C, and (**c**) 400 °C.

Conventional constitutive models frequently struggle to account for the intricate, nonlinear relationships that arise among strain, strain rate, and temperature in advanced composite materials [39,40]. In contrast, the data-driven SVR model leverages its flexibility and robust generalization capabilities to capture these complexities more accurately. Figure 16a–c presents the flow-stress curves for the AZ80-BN composite at 300 °C, 350 °C, and 400 °C, respectively, across four distinct strain rates (0.01, 0.1, 1, and 10 s^−1^, i.e., 10^−2^ s^−1^ to 10^1^ s^−1^). A direct comparison among the experimental (EXP) data, the SVR model, and the Arrhenius constitutive model (ACM) reveals that the SVR-based predictions track the experimental trend more consistently for all strain rates. While both the ACM and SVR models capture the overall transition from initial work hardening to subsequent flow softening, the ACM tends to show noticeable deviations at higher strain rates (1 and 10 s^−1^) and elevated temperatures, suggesting it lacks the necessary flexibility to accurately represent dynamic recrystallization (DRX) and other complex mechanisms at play. By contrast, the SVR approach, relying on pattern recognition rather than fixed functional forms, effectively learns the complex constitutive response from the experimental data. This advantage is especially evident in the peak-stress region and the ensuing flow-stress decline, where the SVR model remains closely aligned with the EXP data at strain rates as high as 10 s^−1^. In this way, the SVR method demonstrates a superior capability to predict the combined effects of strain, strain rate, and temperature, ultimately facilitating a more robust and accurate characterization of AZ80-BN under diverse thermomechanical conditions. Overall, these findings underscore the SVR model’s potential as a powerful, data-driven tool for predicting the flow-stress behavior of advanced composite materials, outperforming traditional, equation-based approaches such as the ACM, particularly in settings where large variations in strain rate and temperature drive complex microstructural evolution.

## 4. Conclusions

This work presents a comprehensive investigation into the thermal deformation behavior of the AZ80-BN magnesium composite, integrating experimental methods, constitutive modeling, and machine learning. The addition of boron nitride as a reinforcing agent significantly enhances high-temperature performance, evidenced by higher peak stress and improved microstructural stability. Dynamic recovery and recrystallization were identified as key mechanisms influencing the overall flow behavior.

Although the Arrhenius-type equation effectively captures peak flow stress, the Support Vector Regression (SVR) model excels in predicting complex, nonlinear dependencies, achieving an *R*^2^ value of 0.9993 and an *AARE* of 0.6321%. In parallel, three-dimensional thermal processing maps pinpoint optimal deformation conditions and potential instability zones, providing valuable guidance for refining processes to produce defect-free components.

These findings underscore the advantage of combining data-driven models with traditional approaches, accelerating materials design, and optimizing processing parameters. Future research should extend this framework to other material systems and integrate microstructural descriptors, further improving model accuracy and enabling intelligent manufacturing of lightweight, high-performance alloys for next-generation engineering applications.

## Figures and Tables

**Table 1:** Mean squared error (MSE) for different models using normalized features across various random states.

Random State	Linear MSE	Polynomial MSE	RBF MSE
42	571.4193	381.1453	207.6735
100	571.1523	381.9096	207.3575
123	571.3632	380.7088	206.3911
321	571.1446	379.9889	208.0404
520	571.9399	380.4232	208.7133
777	570.6064	379.7242	206.5523
888	571.3767	382.1493	209.3180
999	571.3809	380.8332	207.8200
1010	570.6864	380.9306	207.1378
2023	571.2677	380.9256	207.2170
3000	570.7655	382.2015	206.7500

## Data Availability

Data will be made available on request.
